# Dropping the mask: It takes two

**DOI:** 10.1177/13623613231183059

**Published:** 2023-07-05

**Authors:** Julia M Cook, Laura Crane, William Mandy

**Affiliations:** 1 University College London, UK; 2 King’s College London, UK

**Keywords:** adults, autism spectrum disorders, camouflaging, qualitative research, social cognition and social behaviour

## Abstract

In some social situations, autistic people feel pressure to modify their innate social behaviour (i.e. camouflage), while in other social situations they feel free to engage in ways that feel authentic or true to themselves. To date, the latter aspect of autistic people’s experience has rarely been explored. Using an online qualitative survey, this study examined 133 autistic people’s experiences and perspectives of socialising in ways that felt authentic to them, with a particular focus on mixed-neurotype interactions and the role of nonautistic people. Using reflexive thematic analysis, four themes were generated: (1) embracing diverse communication styles, interests and perspectives; (2) creating a more inclusive mixed-neurotype social environment together; (3) minimising and managing mixed-neurotype miscommunication in mutually beneficial ways; and (4) enjoyable interactions involving reduced anxiety and exhaustion as well as genuine connection and rapport. These findings are discussed with reference to theory and research involving the construct of authenticity both inside and outside the field of autism research. The knowledge generated in this study illuminates a previously underexplored aspect of autistic people’s experience and elucidates potential avenues through which to enhance the social experiences and well-being of this group.

**Lay abstract:**

In some situations, autistic people feel pressure to change their social behaviour by camouflaging. In other situations, autistic people feel they don’t need to change their social behaviour. Instead, they feel they can socialise in ways that feel authentic or true to themselves. Past research has tended to focus on autistic people’s experiences of camouflaging rather than their experiences of authenticity. In this study, we asked autistic people what it is like for them when they can socialise in ways that feel authentic or true to themselves. Autistic people described authentic-feeling socialising as more free, spontaneous and open than camouflaging. In supportive environments, this kind of socialising had more positive and less negative consequences than camouflaging. Autistic people felt that having self-awareness and acceptance of their own social needs and being around autistic and nonautistic people who were accepting and understanding helped them to socialise in authentic-feeling ways. Autistic people also spoke about communication behaviours they felt nonautistic people should use to help overcome misunderstandings and create autism-friendly social environments. These findings suggest it is helpful for autistic people to have access to supportive and accepting social environments in which they feel able to socialise in ways that feel authentic to them. In creating such social environments, it is important to focus on nonautistic people’s knowledge and attitude towards autistic people and also their ability to use helpful communication behaviours.

## Introduction

When being authentic,^1^ a person is intentionally behaving in a way that aligns with their ‘true self’. In this context, one’s ‘true self’ reflects one’s innate tendencies and inclinations as demonstrated via their beliefs, values, motives, needs, preferences, feelings, self-perception and worldview (Jongman-Sereno & Leary, 2018; Kernis & Goldman, 2006; Leary, 2003; Wood et al., 2008). Prior research suggests that some autistic people associate camouflaging (also referred to as masking, compensating or adaptive morphing; e.g. Lawson, 2020; Pearson & Rose, 2021) with subjective feelings of inauthenticity, alongside negative emotions and experiences (Hull et al., 2017, 2021). In contrast, some autistic people associate socialising behaviours characterised by a reduction or absence of camouflaging with subjective feelings of authenticity, as well as positive emotions and experiences (Chapman et al., 2022; Cook et al., 2021). To date, research in this area has predominately focused on autistic people’s experiences of camouflaging. Here, in contrast, we present data from a sample of autistic adults recruited online about their experiences and perspectives of what we term ‘authentic-feeling socialising’ versus camouflaging, with a particular focus on mixed-neurotype interactions (i.e. where one partner in the interaction is autistic and one is not).

Authenticity is a key issue for groups with concealable stigmatised identities (e.g. LGBTQ+ or Disabled people) who regularly experience social devaluation across multiple interpersonal contexts (Goffman, 1963; Link & Phelan, 2001; Ryan & Ryan, 2019). These groups are frequently compelled to conceal and/or portray a surface presentation of their true self, to secure social acceptance and to avoid stereotyping, prejudice and discrimination. For example, individuals with concealable stigmatised identities may use impression/stigma management strategies to conceal their identity and pass as a member of the dominant nonstigmatised group (*passing*; Goffman, 1963); disclose but downplay the expression of their identity, so as to be appear more ‘palatable’ to the dominant nonstigmatised group (*covering*; Yoshino, 2007); and/or disclose but conceal any identity-related needs from the dominant nonstigmatised group (Vickers, 2017).

Autism can be conceptualised as a stigmatised identity that, similar to some other stigmatised identities (e.g. mental health difficulties; Quinn et al., 2004), exists on a continuum from conspicuous to concealable depending on an individual’s particular profile (Botha & Frost, 2020; Pearson & Rose, 2021). Here, we conceptualise camouflaging as a form of impression/stigma management that is consciously or unconsciously used by autistic people to hide or change autistic characteristics, so as to promote positive and avoid negative impressions of the self in nonautistic others (although we acknowledge that competing conceptualisations of camouflaging exist; Ai et al., 2022; Cage et al., 2022; Miller et al., 2021; Perry et al., 2022). Specific camouflaging strategies used by autistic people are diverse, but common examples include suppressing repetitive hand movements, forcing facial expressions, avoiding discussion of specialised interests, using conversational scripts and feigning social understanding (Cook et al., 2022). Camouflaging strategies may differentially operate within social interactions, by for example hiding autistic characteristics (masking) or compensating for autism-related social difficulties (compensation; Ai et al., 2022; Hull et al., 2017; Livingston et al., 2019). Autistic people who have not disclosed their autistic identity may use camouflaging strategies to pass as nonautistic, while autistic people who have disclosed their autistic identity may use camouflaging to downplay their autistic differences or reduce the visibility of their autistic needs. Camouflaging is often associated with subjective feelings of inauthenticity; for example, some autistic people describe differences between their true behaviours and their camouflaging behaviours, likening the latter to acting, performing or playing a role (Hull et al., 2017; Livingston et al., 2019).

For groups with concealable stigmatised identities, inauthentic self-presentation can be socially adaptive in reducing stereotyping, prejudice and discrimination, but can be simultaneously harmful to personal relationships and well-being (Ryan & Ryan, 2019). Qualitative research about autistic people’s experiences indicates that camouflaging is associated with negative intrapersonal and interpersonal consequences. Some autistic people feel that, over time, camouflaging interferes with identity formation and results in an uncertain or unstable sense of self (e.g. Bargiela et al., 2016; Bradley et al., 2021; Livingston et al., 2019; Miller et al., 2021). Others report that camouflaging threatens their self-perception and results in negative self-directed emotions and attitudes related to feeling fake or deceptive (Hull et al., 2017). Similarly, some suggest that engaging in camouflaging reduces feelings of connection and closeness in social relationships and, as a result, exacerbates feelings of social isolation and loneliness (Cook et al., 2021; Hull et al., 2017).

Camouflaging is often framed as essential for achieving pragmatic and relational goals in some contexts but not others (e.g. Bradley et al., 2021; Cage & Troxell-Whitman, 2019; Hull et al., 2017; Livingston et al., 2019). Across several qualitative studies, autistic people reported that camouflaging is not necessary when communicating either with other autistic people (Crompton et al., 2020; Howard & Sedgewick, 2021), with accepting nonautistic people (Howard & Sedgewick, 2021) or with established social partners (Hull et al., 2017). Such social interactions, which are characterised by a lack of, or reduction in, camouflaging, are sometimes perceived by autistic people as feeling more authentic and associated with increased positive emotions (e.g. ease and enjoyment) and decreased negative emotions (e.g. stress and anxiety; Cook et al., 2021; Crompton et al., 2020). Owing to the early nature of these findings and a lack of existing research specifically focused on authenticity, further, more targeted exploration is required. In this study, we examine the experiences of a group of autistic people recruited online, comparing and contrasting their experiences and perspectives of ‘authentic-feeling socialising’ with their experiences and perspectives of camouflaging. We were particularly interested in their experiences within mixed-neurotype interactions, as well as in the role of nonautistic social partners.

## Method

### Participants and recruitment

Participants were recruited via the Cambridge Autism Research Database (CARD; www.autismresearchcentre.net). Individuals were eligible to take part in our online survey if they met the following inclusion criteria: (1) aged more than 18 years, (2) formally diagnosed as autistic by a health care professional and/or multidisciplinary team, and (3) living in the United Kingdom.

One hundred and seventy-eight people engaged with the survey: 133 (74.7%) completed all questions, forming the current sample. Of the 133 participants, 58 (43.6%) identified as women, 57 (42.9%) as men, 12 (9%) as nonbinary or used other gender terminology, and 6 (4.5%) preferred not to say. Of those who reported both their sex and gender (*n* = 126), 15 (11.3%) identified with a gender that differed from their sex designated at birth. Participants’ ages ranged from 18 to 74 years (M = 46.15, SD = 15.67) while age at diagnosis ranged from 3 to 68 years (M = 38.55, SD = 16.93). The Autism Quotient-10 Item (AQ-10) was used to give an estimate of autistic traits within the sample (Allison et al., 2012), and 118 participants (88.7%) scored in the clinical range of 6 or above (M = 8.08, SD = 1.97). Most participants were White, university educated and currently engaged in employment or study. Most indicated a preference for identity-first language but a sizable minority (*n* = 50, 37.6%) preferred person-first language or other terminology. Endorsement of co-occurring conditions or mental health diagnoses was common. See Table 1 for further details.

**Table 1.** Participant characteristics including gender, ethnic group, educational qualifications, occupation, co-occurring conditions, mental health diagnoses and terminology preferences.

	*N* (%)
Gender	
Women	58 (43.6)
Men	57 (42.9)
Nonbinary or other terminology	12 (9)
Preferred not to say	6 (4.5)
Ethnic group	
White	
White English/Welsh/Scottish/Northern Irish/British	100 (75.2)
White Irish	1 (0.8)
Other White background	18 (13.5)
Black/African/Caribbean/Black British	
Black Caribbean	1 (0.8)
Asian/Asian British	
Indian	1 (0.8)
Mixed/Multiple Ethnic Groups	
White and Black Caribbean	2 (1.5)
White and Black African	1 (0.8)
White and Asian	2 (1.5)
Other mixed/multiple ethnic background	5 (3.8)
Other ethnic group	
Any other ethnic background	2 (1.5)
Education	
No qualifications	4 (3.0)
GCSE (school-based, 14–16 years)	8 (6.0)
A levels (school-based, 16–19 years)/level 3 or 4 diploma/foundational degree	25 (18.8)
University education (undergraduate or postgraduate)	91 (68.4)
Other	5 (3.8)
Occupation	
In paid employment (full or part time)	59 (44.4)
In voluntary employment	7 (5.3)
Not employed but looking for employment	10 (7.5)
Unable to work due to disability or illness	21 (15.8)
Full-time carer	3 (2.3)
Retired	15 (11.3)
Studying	8 (6.0)
Other	10 (7.5)
Co-occurring conditions (lifetime)	
Intellectual or learning disability	16 (12)
ADHD/ADD	28 (21.1)
Hearing impairment	13 (9.8)
Vision impairment	12 (9.0)
Physical disability	12 (9.0)
Medical or chronic health condition	27 (20.3)
Genetic condition	8 (6.0)
Other conditions	23 (17.3)
Mental health diagnoses (lifetime)	
Mood disorder	64 (48.1)
Anxiety disorder	66 (49.6)
Addictive disorder	2 (1.5)
Eating disorder	17 (12.8)
Personality disorder	13 (9.8)
Schizophrenia	2 (1.5)
Other mental health condition	10 (7.5)
Terminology preference	
Autistic person	83 (62.4)
Person with autism	29 (21.8)
Other terminology	19 (14.3)
Preferred not say	2 (1.5)

*Note*. Percentages may not sum to 100% because of rounding. Co-occurring conditions and mental health diagnosis categories are not mutually exclusive. Other terminology included terminology such as ‘Asperger’, ‘Aspie’ and ‘neurodivergent’. A range of other conditions were reported by participants under ‘Other Conditions’ included conditions such as stammer, tinnitus and chronic fatigue syndrome. GCSE = General Certificate of Secondary Education; ADHD = Attention Deficit Hyperactivity Disorder; ADD = Attention Deficit Disorder.

### Procedure

Ethical approval was obtained from University College London Research Ethics Committee. Individuals on the CARD were invited to take part via an email containing relevant information about the study and a link to the online survey. Upon following the link, participants read a participant information sheet, provided informed consent and completed the survey. After completing the survey, participants could choose to enter a prize draw to win an iPad.

### Survey development

The survey was developed specifically for this study, in consultation with members of the autistic community. Initially, one autistic person gave their opinion regarding the proposed study including research aims, questions and methods. The authors developed an initial set of questions, which an autistic researcher provided informal feedback on. Next, questions were further developed, refined and finalised based on information gathered during semistructured cognitive interviewing with six autistic people (four women and two men). Cognitive interviewing is a qualitative methodology used to assess survey question performance by exploring: (1) constructs considered by participants in answering questions; (2) if and why participants experience difficulties answering questions; and (3) if and why particular participants interpret questions differently (Willson & Miller, 2014).

The protocol for cognitive interviews (provided in Supplemental Material B) was developed based on prior research with nonautistic adults (Willis, 2005). Each autistic person was interviewed individually by JC via Microsoft Teams for approximately 90 min. Using screen share, interviewees were shown each survey instruction and survey question in turn. They were instructed to read each instruction or question silently, and then answer the question aloud. Based on their responses, interviewees were asked a range of follow-up questions to identify any potential issues with the survey and potential solutions. Each autistic person interviewed was reimbursed for their time.

The final qualitative survey comprised open- and closed-ended questions regarding participants’ experiences of socialising in ways that felt more or less authentic to them (see Supplemental Material C for a full copy of the survey). While the primary focus of this qualitative study was the responses to open-ended questions, closed-ended questions were also used for two reasons. First, one closed-ended question (‘Do you ever camouflage when interacting with other people?’) was included to enable us to customise open-ended questions for participants who did or did not engage in camouflaging (described below). Second, other closed-ended questions were designed as prompts for subsequent open-ended questions. This question format, implemented based on feedback from autistic people during the cognitive interviewing process, was incorporated to increase accessibility.

Participants who reported engaging in camouflaging (*n* = 124) were asked five questions about their experiences of camouflaging (i.e. their awareness of their camouflaging, the frequency of their camouflaging and changes in the frequency of their camouflaging). These participants then completed 12 questions about their experiences of engaging with others in ways that felt more authentic to them (i.e. asking what more authentic-feeling socialising looks like, any differences between camouflaging and more authentic-feeling socialising, any benefits and risks associated with more authentic-feeling socialising, and any factors that enable more authentic-feeling socialising). Participants who stated that they did not engage in camouflaging (*n* = 9) completed eight questions about their experiences of not camouflaging (i.e. what this looks like, any benefits and risks associated with not camouflaging, and any factors that enable them to not camouflage). The survey, in addition, included closed-ended questions to collect participant demographics, as well as the 10-item AQ (Allison et al., 2012).

### Data analysis

Survey responses were analysed thematically within a critical realist framework (Maxwell, 2012) following the reflexive thematic analysis approach developed by Braun and Clarke (2006, 2013, 2022). Thematic analysis is a theoretically flexible approach that can facilitate inductively developed analysis involving both semantic (surface) and latent (implicit) meaning in the data set. This approach is helpful in examining a complex social phenomenon that is located within a wider social system, yet also arises from and impacts upon an individual’s internal experiences.

The analytic process was recursive and involved data familiarisation, coding, theme development and review. As is usual practice in reflexive thematic analysis, one author (JC) completed the coding process (Braun & Clarke, 2022). She read and re-read all survey responses, noting down and reflecting on her initial thoughts and reactions. Next, using NVivo 12, she conducted two codings of the data set; generating and revising codes based on concepts and meanings she identified in the data. The output of the coding stage of analysis included code names, code descriptions and extracts of data within each code. Using this output, JC, WM and LC mapped codes together based on shared meaning to form candidate themes. Then, JC generated candidate theme maps, names and descriptions. Candidate themes were recursively returned to and revised by reviewing theme maps, names and descriptions along with code names, descriptions and data. In the early stages of theme development, this process involved all authors meeting periodically to review all information about themes. Later, in the more final stages of theme review, this process involved authors providing feedback on written drafts of the results. After each review, JC incorporated the views and feedback of all authors and made corresponding amendments to the themes (e.g. re-organising codes, changing descriptions of themes and changing theme names). This process was repeated until all authors reached consensus on the final themes.

Regarding positionality, all authors identify as nonautistic and align with neurodiversity-informed understandings of autism over more medical approaches. The ways in which the authors’ prior knowledge, assumptions and experiences influenced the analysis (as well as the study more broadly) were interrogated via reflexive journaling, bracketing interviewing (Fischer, 2009) and group discussion.

### Member reflections

To ensure results were reported in an ethical and respectful manner (Braun & Clarke, 2022), 10 participants (who participated in the survey) provided feedback on a version of the near-final themes, written in lay language. These participants were reimbursed for their time. Based on this feedback, a synonym for the term ‘authentic’ was removed from the results, because one participant felt it could potentially be associated with harmful stereotypes about autism.

### Community involvement statement

This study was led by a team of nonautistic researchers, so we were keen to include autistic input. While it was not possible to involve autistic people at all stages of the research (due to time/resource constraints), autistic people were consulted during the formation and design of the study and prior to dissemination of findings (as detailed above).

## Results

**Figure 1.**
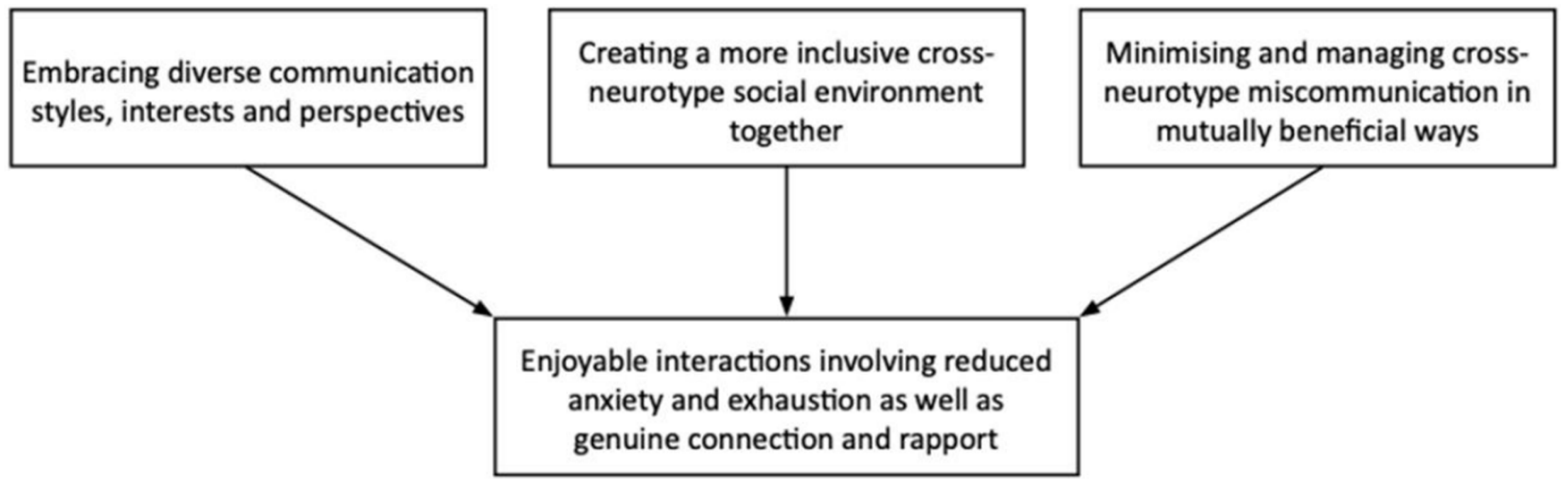
Overview of themes.

In the following four themes, we present participants’ perspectives of socialising in ways that felt authentic to them, within the context of safe, comfortable and accepting social relationships. As seen in Figure 1, authentic-feeling socialising was characterised by (1) embracing diverse communication styles, interests and perspectives; (2) creating a more inclusive mixed-neurotype social environment together; (3) minimising and managing mixed-neurotype miscommunication in mutually beneficial ways; resulting in (4) enjoyable interactions involving reduced anxiety and exhaustion as well as genuine connection and rapport. Illustrative quotes for each theme are provided in the text. Participants’ gender and age appear after quotes.

### Embracing diverse communication styles, interests and perspectives

From childhood or adolescence, participants described being aware that their communication styles, interests and perspectives were often different from their peers. Self-acceptance of one’s differences was often associated with feeling ‘allowed’ or ‘permitted’ to engage in ways that felt more authentic:

I started accepting myself more which translated into allowing myself to be myself more. (Woman, aged 50)

Diagnosis was often described in relation to increasing self-acceptance. Before having an autism diagnosis, some participants viewed their autistic characteristics and traits as indicative of personal failure or even flawed character. The validation and explanation of their differences afforded by a diagnosis allowed some participants to challenge these negative self-conceptualisations and improve their self-acceptance and confidence:

Since my diagnosis I feel like I am not bad or stupid or an alien so I should just be me. (Woman, aged 30)

However, it appeared that self-acceptance alone was not sufficient in enabling interactions that felt authentic. Rather, a mutual understanding that multiple, valid communication styles exist was seen as essential in enabling authentic-feeling interactions. Participants spoke of wanting nonautistic social partners specifically to understand and accept differences in autistic and nonautistic communication and to refrain from applying nonautistic interpretations to autistic communication. Participants valued nonautistic social partners who refrained from criticising, commenting on or making fun of autistic communication:

Accept that there are a multitude of communication ‘styles’, that theirs [non autistic people’s] is not the default, and that people that may deviate from theirs are not, automatically, without doubt, being rude. (Man, aged 58)

Participants spoke of the importance of nonautistic social partners not explicitly or implicitly ‘encouraging’, ‘expecting’ or ‘insisting’ autistic people use nonautistic social behaviours. For example, one participant explained:

Do not demand eye contact even in non-verbal ways. (Woman, aged 47)

Participants reflected that with such mutual understanding and acceptance (within same or mixed-neurotype contexts), they reduced the extent to which they monitored and censored themselves in interactions. Instead, they engaged in a more ‘spontaneous’, ‘open’ and ‘free’ manner and used more comfortable levels of eye contact, directness (e.g. ‘shorter, more direct responses to questions’ [Man, aged 58]), reciprocity (e.g. ‘talk as or when I want’ [Man, aged 33]) or repetitive movements (e.g. ‘stim by making noises, tapping my fingers on my hand or fidgeting’ [Woman, aged 31]). As one participant explained:

It allows me some (not total) relaxation of the self-monitoring, such that I am able to let out all of the stuff I have been actively restraining under the tightly-laced ‘suitable behaviour’ corsets, often for a period of several, or many, weeks. (Man, aged 60)

A mutual openness to, and acceptance of, differing interests, perspectives and sense of humour was, in addition, viewed as essential in enabling socialising that felt authentic. With such openness and acceptance (within same or mixed-neurotype contexts), participants spoke more freely about their interests and hobbies, shared their opinions and showed their sense of humour:

I talk more about things I am interested in, which I might be too embarrassed to do with other people and I make really bad jokes. (Woman, aged 32)

### Creating a more inclusive mixed-neurotype social environment together

Participants possessed a strong awareness of their distinctive social needs and preferences as well as the way predominately nonautistic environments could be adapted to better suit these. As in the previous theme, gaining an autism diagnosis increased some people’s understanding and acceptance of their social needs and preferences, as well as potentially helpful and unhelpful coping strategies. However, a small number of participants described difficulties engaging in ways that felt authentic to them owing to difficulties discerning their own needs:

The trouble is that I am so used to doing what others want that I nearly always (99%) go along with what others want. For them to start considering me and what I want would leave me at a loss as I am no longer sure what I would want. (Man, aged 61)

Many participants considered and arranged environmental adaptations they required prior to mixed-neurotype social interactions. For example, participants described asserting their social needs and preferences by choosing to socialise in certain environments, with a certain number of people, for a certain length of time:

In my social life, I keep meetings on my terms – places I feel relaxed, quiet, comfortable – I plan everything. (Nonbinary person, aged 40)

Participants also communicated or asserted their social needs and preferences during mixed-neurotype social interactions as necessary with.

I ask for sounds to be turned down, for example my partner’s mum always has the radio on when we visit and I always ask for it to be turned down or I can’t engage in conversation because it bothers me to have noise in the background. (Woman, aged 32)

In creating an inclusive mixed-neurotype social environment, participants emphasised the importance of nonautistic social partners understanding and accepting autistic ways of being in, and experiencing, the world. Participants valued nonautistic social partners who listened to and empathised with their experiences, especially their unique difficulties:

Listen, and let me explain. Accept my explanation. (Woman, aged 56)

Participants also wanted nonautistic people to actively participate in this process by asking participants about their difficulties and needs:

Ask me what I need. Ask what they [non autistic social partners] can do to help. (Agender person, aged 27).

In addition, participants spoke of the importance of nonautistic social partners respecting boundaries. Participants wanted nonautistic people to refrain from, ‘persuading’, ‘pushing’ or ‘cajoling’ them to go beyond their limits:

Accept it if I say I’m tired and should go home at 10pm, instead of trying to talk me out of it. (Woman, aged 37)

### Minimising and managing mixed-neurotype miscommunication in mutually beneficial ways

Participants spoke of their difficulties understanding the social communication and expectations of nonautistic social partners. Often, when engaging in ways that felt authentic to them, participants sought to gain understanding immediately by asking nonautistic others for clarification or feedback:

I’m able to say if I don’t understand something that’s happened, or if they’re making a facial expression that doesn’t make sense to me, or if I don’t get a joke. (Woman, aged 40)

In responding to such requests, participants highlighted the importance of nonautistic social partners being amenable to providing additional or alternative explanations:

Be generous with your time and information if I ask please to explain things. (Nonbinary person, aged 39)

Participants further reflected that their challenges in understanding nonautistic social partners were reduced when nonautistic social partners were clear and explicit in communicating their thoughts, feelings and intentions. For example, one participant explained that nonautistic social partners should:

[Avoid] using unclear language or relying solely on body language to get a message across. (Man, aged 58)

At the same time, participants’ accounts suggested that nonautistic social partners also experienced difficulties understanding participants’ social expectations and communication. In these instances, participants felt it was important that nonautistic social partners held them in positive regard if feeling confused by their specific behaviours:

Take my interactions at face value and work with the default assumption I am honest and well-intentioned, not that there is a hidden meaning to anything I say, or that I am deliberately rude or [I] think badly of them if I don’t react in the way they necessarily expect. (Man, aged 28)

Participants also reported that it was helpful for nonautistic social partners to avoid making assumptions and instead ask for clarification:

Ask me for clarification if something I say or do doesn’t make sense to them, instead of making an assumption that might hurt our relationship. (Woman, aged 28)

### Enjoyable interactions involving reduced anxiety and exhaustion as well as genuine connection and rapport

Authentic-feeling social interactions within the context of safe, comfortable, understanding and accepting same or mixed-neurotype contexts were described as positive experiences that participants enjoyed rather than ‘endured’:

It’s like being set free, in a way. Not having to pretend. Sometimes, one can even have fun! (Man, aged 63)

When engaging in ways that felt more authentic to them, participants also described feeling ‘more relaxed’ and ‘less anxious’, or ‘less stressed’. Participants associated these emotional improvements with reductions in the sense of pressure and expectation they felt to conform socially (e.g. ‘less stress and anxiety to try to conform and fit in’ [Man, aged 57]), their use of camouflaging behaviours, and fears they held about being exposed as a social outsider (e.g. ‘being more relaxed and not being scared that the camouflage will be lifted somehow’ [Woman, aged 56]).

However, a few participants spoke of continuing to experience anxiety when engaging in ways that felt more authentic to them, owing to fears of negative interpersonal outcomes. These participants appeared to be particularly attuned to risks of socialising in ways that felt authentic to them. As one participant explained:

[I] worry at times afterward about how I appeared. (Woman, aged 45)

Socialising in ways that felt authentic was described as less cognitively demanding and exhausting than camouflaging:

It doesn’t require constant concentration and high levels of energy. (Woman, aged 50)

As a result, when engaging in ways that felt more authentic to them, some participants felt they had increased capacity to focus and engage with.

I’m able to devote more of my mental energy to whatever I’m supposed to be doing rather than spending most of my time thinking about how autistic I appear so I’m able to perform better in academic contexts and to listen better and respond more fully in social contexts. (Man, aged 30)

Other participants described an increased capacity to cope with day-to-day challenges or difficulties that arose:

When not masking, I am able to deal better with challenges such as something unexpected. I attribute this to having the spare brainpower to do so. (Woman, aged 30)

Authentic socialising also appeared to strengthen participants’ personal relationships. Participants described authentic-feeling socialising as improving their ability to form more ‘genuine’ connections and rapport with others who appreciated and valued them for their true selves with.

Being authentic also gives me a sense of connectedness and helps to foster friendships because I am revealing my true self rather than a rather boring mask, so the people who like the real me will gravitate towards me. (Man, aged 28)

## Discussion

In this study, we present data from a sample of autistic adults recruited online, comparing and contrasting their experiences and perspectives of authentic-feeling socialising versus camouflaging, with a particular focus on mixed-neurotype interactions as well as the role of nonautistic social partners. We found that most participants engaged in camouflaging. However, within some social relationships, many (but not all) participants experienced enjoyable and satisfying interactions in which they engaged in ways that felt authentic to them. Such experiences typically involved autistic and nonautistic friends, family or romantic partners who demonstrated qualities such as understanding and acceptance. Participants further described their own self-acceptance and awareness as factors enabling authentic-feeling socialising. In this way, participants’ experiences of socialising in ways that felt authentic to them appeared best understood as an interpersonal process, dependent on the actions of all social partners involved. Next, we discuss key features of our four identified themes with reference to research on camouflaging as well as the broader literature on authenticity and stigma.

### What feels authentic?

In line with previous qualitative research (Chapman et al., 2022; Cook et al., 2021; Crompton et al., 2020; Howard & Sedgewick, 2021; Schneid & Raz, 2020), participants commonly described engaging in specific authentic-feeling behaviours or processes; many of which appeared to contrast with camouflaging. Of note, participants described decreasing their self-monitoring and censoring; increasing their self-disclosure; enacting more comfortable (and seemingly more autistic) levels of eye contact, directness, reciprocity and repetitive movements; openly communicating any social difficulties or confusion; and asserting their social needs and preferences. These findings suggest that for participants in this study, enacting their autistic identity (e.g. engaging in autism-congruent behaviours, making autism-related self-disclosures and asserting autism-related needs) during interpersonal interactions felt authentic. Such experiences are consistent with broader research on felt authenticity, which demonstrates that for people with stigmatised identities, identity enactment facilitates felt authenticity whereas identity concealment impedes felt authenticity (e.g. Crabtree & Pillow, 2020; Newheiser & Barreto, 2014).

### Benefits of socialising in ways that feel authentic

Participants reported that socialising in ways that felt more authentic to them was associated with more positive interpersonal and intrapersonal consequences than camouflaging. Specifically, social behaviours that felt authentic were associated with increased feelings of relaxation and decreased feelings of anxiety and stress; reduced feelings of cognitive exhaustion and increased capacity to focus, engage and manage day-to-day stressors; and increased feelings of interpersonal connection and rapport. These findings align with extant literature conducted with the general population, demonstrating that felt authenticity is positively associated with positive emotions (particularly contentment and relaxation; Lenton et al., 2013) and more satisfying, higher quality social relationships (Brunell et al., 2010; Le & Impett, 2013 Peets & Hodges, 2018); but also negatively associated with mental exhaustion (Huppertz et al., 2020; van den Bosch & Taris, 2014, 2018).

### Intrapersonal factors and authentic-feeling socialising

Knowledge and acceptance of one’s propensities and characteristics (i.e. one’s true self) and consequent expression of these propensities and characteristics are thought to be foundational to authenticity (Kernis & Goldman, 2006). Consistent with this view, participants’ accounts suggested that having awareness and acceptance of social needs and preferences, along with skills in effectively communicating and asserting these needs and preferences, enabled them to socialise in ways that felt authentic.

For some, but not all, participants, gaining an autism diagnosis was seen as enhancing the development of self-awareness and acceptance as well as self-advocacy skills. However, it is important to note that it was often unclear if such positive effects related to the provision of a diagnostic label (and access to information, support and community, for example) or the actual diagnostic process per se. Indeed, prior research suggests that autistic people are often dissatisfied with the latter (e.g. Crane et al., 2018; Jones et al., 2014; Lewis, 2017). Regardless, these insights add to extant literature demonstrating the importance of access to timely diagnosis in improving the lives of autistic people (Bargiela et al., 2016; Crane et al., 2018; Huang et al., 2020; Lilley et al., 2021; Mandy et al., 2022; Zuckerman et al., 2014).

### Interpersonal factors associated and authentic-feeling socialising

Importantly, our findings suggest that most participants engaged in authentic-feeling socialising within the context of safe, comfortable and accepting relationships; and that the benefits of authentic-feeling socialising appeared to be specific to these relationships. These findings echo an extensive body of interpersonal research outside the field of autism, highlighting the central role of social contexts in facilitating and supporting authenticity, especially for those with stigmatised identities (Ryan & Ryan, 2019). Supportive social contexts facilitate the development of individual-level processes required for authenticity (e.g. self-knowledge, self-acceptance and identity integration; Weinstein et al., 2017). Supportive social contexts also enable stigmatised individuals to meet their psychological needs for belonging and acceptance (as well as providing associated psychological benefits e.g. positive affect) without resorting to an inauthentic self-presentation (Leary, 2003).

Regarding features of a supportive social context that facilitate authentic identity exploration and expression, as well as feelings of belonging and acceptance for autistic people, participants in this study emphasised the importance of nonautistic people’s use of communication behaviours. Specifically, participants described the importance of nonautistic people being able to seek information about other people’s communication styles, needs and preferences (e.g. asking questions and active listening); nonautistic people understanding their own social communication style, needs and preferences and perceiving the impact of these upon others (e.g. reflection and monitoring); and nonautistic people adapting their communication accordingly (e.g. being more explicit or reducing reliance on nonverbal communication). These accounts highlight the influence of bi-directional differences in social communication style and reciprocal challenges in understanding on the quality of mixed-neurotype interactions (i.e. the double empathy problem; Milton, 2012). In addition, while a dearth of research examines mixed-neurotype communication skills required by nonautistic people in facilitating mutually satisfying interactions with autistic people, the current findings are in line with a small body of qualitative research suggesting autistic people view nonautistic people’s ability to use direct, open and clear communication as important factors (Brownlow et al., 2021).

Improvements in the social experiences and well-being of autistic people will likely be facilitated via access to supportive social contexts in which they can authentically express their autistic identity (Cage & Troxell-Whitman, 2019). Regarding cross-neurotype social contexts specifically, there is a need for initiatives aimed at improving nonautistic people’s ability to relate to autistic people. Such interventions should target both nonautistic people’s knowledge about and attitudes towards autistic people, as well as nonautistic people’s cross-neurotype communication skills. Exploring existing frameworks and interventions that aim to improve communication between different cultural and other social groups may be useful in developing such interventions (e.g. Arasaratnam, 2012; Hagqvist et al., 2020; Rasmussen & Sieck, 2015).

It is also important to acknowledge that our sample of predominately White, university educated and employed autistic people may experience less stigma and have more access to supportive social contexts than some other groups within the autistic community. It is imperative that future research examines the experiences of authenticity for autistic people with multiple stigmatised identities (e.g. autistic Black, Indigenous or people of colour [BIPOC]) that encounter multiple and compounded forms of stereotyping, prejudice and discrimination and for whom authentic-feeling socialising may currently be dangerous (Jones et al., 2020).

### Strengths and limitations

This study is strengthened via community-engaged practices. Specifically, autistic people were consulted at multiple stages of the project including during formation and design of the study, as well as prior to dissemination. Such consultation enhanced the real-world relevance and validity of the study and findings, improved the accessibility of research methods and quality of data collected, and ensured ethical, respectful and effective dissemination. However, due to time/resource constraints, the study is limited by the absence of autistic input during data analysis.

The quality of the thematic analysis was ensured via reflexive, systematic and extended engagement with the data (Braun & Clarke, 2022). Specifically, analysis was conducted over an extended period of 4 months; the ways in which the authors’ prior knowledge, assumptions and experiences influenced the analysis were interrogated via reflexive journaling, bracketing interviewing and group discussion; and interpretation was deepened via collaborative engagement in analysis. However, involving an autistic collaborator would have illuminated an additional and important perspective on the data, thereby deepening the interpretation further.

Finally, and as previously discussed, the findings generated here are based on the social experiences and perspectives of a fairly homogeneous sample of mostly White, university educated and late-diagnosed autistic people; as is common in online survey research with autistic samples (Rødgaard et al., 2022). While the goal of qualitative research is not generalisability, it is important to stress that these results will likely not reflect the social experiences and perspectives of other groups of autistic people, especially those with multiple stigmatised identities. Further research specifically focused on such groups using additional recruitment methods, and offering multiple modes of participation is now needed (Nicolaidis et al., 2019). In addition, given that the inclusion criteria for this study required a formal diagnosis from a health care professional and/or multidisciplinary team, the results (and subsequent recommendations) may not generalise to adults who are autistic, but who are unable to access the resources needed to obtain a formal diagnosis (see Lewis, 2017).

## Conclusion

This study details autistic people’s experiences and perspectives of socialising in ways that feel authentic to them, within the context of safe, comfortable and accepting interactions. Our findings suggest that for autistic people (as for those with other stigmatised identities), authentic-feeling socialising is best understood as an interpersonal process, influenced by the social context. The social experiences and well-being of autistic people will likely be improved via access to supportive social contexts that facilitate authentic identity exploration and expression and fulfil psychological needs for belonging and acceptance.

## Supplemental Material

sj-docx-1-aut-10.1177_13623613231183059 – Supplemental material for Dropping the mask: It takes two Supplemental material, sj-docx-1-aut-10.1177_13623613231183059 for Dropping the mask: It takes two by Julia M Cook, Laura Crane and William Mandy in Autism

## Notes

1. We acknowledge that the language used by researchers can powerfully shape people’s beliefs, understanding and position on a topic in both helpful and unhelpful ways (Lawson, 2020). Please see the statement in the supplemental materials regarding our position and rationale for using the term ‘authenticity’ in this article.
