# Supplementary material for: Dropping the mask: It takes two
Source: Autism. 2023 Jul 5;28(4):831–42. doi: 10.1177/13623613231183059 (PMC10981180; doi:10.1177/13623613231183059)
Supplement: sj-docx-1-aut-10.1177_13623613231183059 – Supplemental material for Dropping the mask: It takes two [file sj-docx-1-aut-10.1177_13623613231183059.docx]

# Supplementary Material A

**Statement on Terminology**

It is important to acknowledge that autistic people have been, and continue to be, harmed by

unhelpful stereotyped views of what autism “looks like” (e.g., Bargiela et al., 2016; Pearson & Rose, 2021). In this study, we do not mean to imply there is a correct, valid, or authentic way of being *autistic*. Rather this study presents an exploration of individuals’ experiences of socialising in ways that feel authentic to *them* as individuals*.* The term authentic was chosen for several reasons. First, this is the term used in psychological literature to describe a subjective experience of congruence between one’s actions and true self. Second, within qualitative research and autistic people’s writings (e.g., Blackwater, 2022; Wiltshire, 2021), this term has been used to describe a social experience that contrasts camouflaging. Finally, a subset of participants in the current study were consulted and provided positive feedback regarding the appropriateness of the term within the context of the study. However, given the early stage of this research field, we acknowledge it is important to continue to learn from the autistic community regarding the usefulness and appropriateness of this terminology.

# Supplementary Material B

**Cognitive Interview Guide**

The following is a semi-structured interview guide that was developed following recommendations set out in Willis (2005). When necessary, the interviewer will ask the interviewee follow up questions to clarify their responses.

1. The interviewer will share their screen with the interviewee. The interviewee will be shown the instructions for the demographic section of the questionnaire.

**Question:** In your own words, can you tell me what the instruction is telling you?

1. Participants will be shown each demographic question in turn. Participants will be instructed to read the question. They will then be asked to answer the question aloud. Next, participants will be asked the following questions:

**Question:** How easy was that question for you to answer (on a scale from 1 to 5, with 1 being very easy, 3 being neither easy nor difficult, and 5 being very difficult)? Can you tell me more about that?

**Question:** Could this question be improved in anyway?

1. The interviewee will be shown the instructions for the qualitative section of the questionnaire.

**Question:** In your own words, can you tell me what the instruction is telling you?

1. Participants will be shown each qualitative question in turn. Participants will be instructed to read the question. They will then be asked to answer it aloud. Next participants will be asked the following questions:

**Question:** Can you tell me in your own words what that question is asking? **Question:** How easy was that question for you to answer (on a scale from 1 to 5, with 1 being very easy, 3 being neither easy nor difficult, and 5 being very difficult)? Can you tell me more about that?

1. If participants’ answers to either (1) the original qualitative question or (2) the above probes

indicate a word or phrase within the question is ambiguous or vague*:*

**Question:** What does the word/phrase (*insert word/phrase*) mean to you as it is used in this question?

1. If participants’ answers to either (1) the original qualitative question or (2) the above probes

indicate the question is not readily comprehensible:

**Question:** How would you word this question or how would you improve this question?

1. If participants’ answers to either (1) the original qualitative question or (2) the above probes

indicate the interviewee thinks the question does not apply to them:

**Question:** How well does that question apply to you? Can you tell me more about that?

1. Once this procedure has been completed for all qualitative questions: **Question:** Did the order of the questions cause you any difficulties? **Question:** Was there any question that caused you to feel offence or distress?

**Question:** Is there anything else you would like to say about the survey questions or the survey generally?

# Supplementary Material C

**Qualitative Survey Questions**

Some autistic people feel they need to change their natural or usual social behaviour when socialising, talking, or interacting with other people in order to fit in, cope, or get by. There are many, many different ways in which autistic people may do this. A few examples include:

- - Stopping one’s stimming hand movements
  - Forcing eye contact even if it feels uncomfortable
  - Changing one’s tone of voice
  - Avoiding talking about one’s hobbies or interests
  - Avoiding talking about oneself all together
  - Using rehearsed or practiced conversational scripts, jokes, or anecdotes

This is sometimes called camouflaging, masking, or passing. In this survey, we will use the term camouflaging to mean camouflaging, masking, or passing.

1. Do you ever camouflage when interacting with other people? (response options = yes/no)

**Definition:** By interacting we mean any situation in which two or more people are communicating (e.g., two or more people talking in person, talking over the phone, or via video calling or communicating by text via instant messaging, texting, or emailing).

1. Overall, how aware of your camouflaging are you? (sliding scale from “I am almost never aware” to “I am almost always aware”). If you prefer, please feel free to explain in your own words.
2. Over the last year, how frequently have you tried to camouflage when interacting with other people? (sliding scale from “almost never” to “almost always”). If you prefer, please feel free to explain in your own words.
3. How as the pandemic impacted your camouflaging over the last year?
4. Over your lifetime, has the frequency with which you camouflage when interacting with others changed? (response options = Yes, overall it had decreased; Yes, overall it has increased; No, overall it has stayed the same; and Other (please describe). If you prefer, please feel free to explain in your own words.

If the frequency of your camouflaging has changed:

1. Can you please tell us more about how your camouflaging has changed over time? We’re

interested to know things like:

- 1. When you started camouflaging
  2. When the frequency of your camouflaging changed
  3. Why the frequency of your camouflaging changed

Some autistic people say that they don’t always change their natural or instinctive social behaviour when interacting with others. They say that around certain people they feel less pressure or need to camouflage. Instead, they feel they can be more like their natural, authentic, or true self.

1. Are there certain people with whom you feel you can be a more like your natural, authentic, or true self? (response options = yes/no). If you prefer, please feel free to explain in your own words.
2. If yes, who are these people? Please note, we do not require the specific names of people, just your relationship to them (i.e. friend, partner, co-worker).
3. Why do you feel you can be more like your natural, authentic, or true self around these people?

When you are with people that you feel like you can you be more like your natural, authentic, or

true self around…

1. What do you do or how do you behave? Please feel free to provide specific details and examples.
2. How is this different to what you do or how you behave when you are camouflaging?

Some (but not all) autistic people report that when they interact with non-autistic people they experience sensory or social difficulties. When you are with non-autistic people with whom you feel like you can be more like your natural, authentic, or true self…

1. What (if anything) do you do about any sensory needs or difficulties you may have? For example, any needs or difficulties you may have related to your sense of hearing, touch, smell, or sight.

 N/A there are no non-autistic people in my life that I can be more like more natural, authentic, or true self around.

1. What (if anything) do you do about any social needs or difficulties you may have? For example, needs or difficulties you may have related to:

- Understanding other people’s verbal communication (e.g., sarcasm, jokes, white

lies, or vague/ambiguous language)

- Understanding other people’s non-verbal communication (e.g., their facial expressions, body language, or gestures)
- Becoming fatigued or tired from socialising
- Interacting with several people at the same time

 N/A there are no non-autistic people in my life that I can be more like more natural, authentic, or true self around.

Overall, in your everyday life……

1. What (if any) are the advantages/benefits of being your natural, authentic, or true self when interacting with others (i.e., not camouflaging)?
2. What (if any) are the disadvantages/risks of being your natural, authentic, or true self when interacting with others (i.e., not camouflaging)?
3. When interacting with you, what should a non-autistic person (e.g. family member, friend, co-worker) do in order to be welcoming, accepting, and/or helpful?
4. When interacting with you, what should a non-autistic person (e.g. a family member, friend, co-worker) avoid doing in order to be welcoming, accepting, and/or helpful?
5. Please feel free to give any other comments below that you may have about:

- camouflaging
- being your more natural, authentic, or true self.

# Alternative Questions

If interviewee selects “No” to Q1 (i.e. the interviewee indicates they have never camouflaged):

Some (but not all) autistic people report that when they interact with non-autistic people they experience sensory or social difficulties. When you are interacting with non-autistic people…

1. What (if anything) do you do about any sensory needs or difficulties you may have? For example, any needs or difficulties you may have related to your sense of hearing, touch, smell, or sight.
2. What (if anything) do you do about any social needs or difficulties you may have? For example, needs or difficulties you may have related to:
   - Understanding other people’s verbal communication (e.g. sarcasm, jokes, white lies, or

vague/ambiguous language)

- - Understanding other people’s non-verbal communication (e.g. their facial expressions, body language, or gestures)
  - Becoming fatigued or tired from socialising
  - Interacting with several people at the same time

1. How would you rate your experiences of interacting with non-autistic people? (likert scale 1-5, with 1 = almost always negative, 3 = neither positive nor negative, and 5 = almost always positive). Please feel free to provide additional comments about your experiences of interacting with non-autistic people.

When interacting with non-autistic people……

1. What (if any) are the advantages/benefits of not camouflaging your autistic characteristics?
2. What (if any) are the disadvantages/risks of not camouflaging your autistic characteristics?
3. When interacting with you, what should a non-autistic person (e.g. family member, friend, co- worker) do in order to be welcoming, accepting, and/or helpful?
4. When interacting with you, what should a non-autistic person (e.g. a family member, friend, co-worker) avoid doing in order to be welcoming, accepting, and/or helpful?
5. Is there anything else you would like to say?

# Supplementary References

Bargiela, S., Steward, R., & Mandy, W. (2016). The experiences of late-diagnosed women with autism spectrum conditions: An investigation of the female autism phenotype. *Journal of Autism and Developmental Disorders, 46*(10), 3281-3294. https://doi.org/10.1007/s10803-016-

2872-8

Blackwater, A. (2022, January 12). How becoming my ‘authentically autistic’ self has helped me. *The Mighty.* https://themighty.com/2022/01/authentically-autistic-identity/

Pearson, A., & Rose, K. (2021). A conceptual analysis of autistic masking: Understanding the narrative of stigma and the illusion of choice. *Autism in Adulthood, 3*(1), 52-60. https://doi.org/10.1089/aut.2020.0043

Willis, G. B. (2005). Developing standard cognitive probes. In Willis, G. B. Cognitive interviewing (pp.

66-86). *Thousand Oaks, CA: SAGE Publications.* <http://10.4135/9781412983655> Wiltshire, R. (2021, June). On being authentically autistic. *Altogether Autism Journal.*

https://issuu.com/lifeunlimited1/docs/altogether_autism_june_2021
